# Serology-Based Screening and Prevalence of Schistosomiasis, Strongyloidiasis, and Chagas Disease in Migrants Living with HIV: Results from a 10-Year Retrospective Cohort in an Italian Tertiary Healthcare Center

**DOI:** 10.3390/tropicalmed10100294

**Published:** 2025-10-16

**Authors:** Anna Barbiero, Riccardo Paggi, Sasha Trevisan, Giuseppe Gasparro, Michele Spinicci, Costanza Malcontenti, Marco Pozzi, Paola Corsi, Gian Maria Rossolini, Maria Grazia Colao, Alessandro Bartoloni, Lorenzo Zammarchi, Filippo Lagi

**Affiliations:** 1 Department of Experimental and Clinical Medicine, University of Florence, 50121 Florence, Italy; anna.barbiero@unifi.it (A.B.); riccardo.paggi@unifi.it (R.P.); sasha.trevisan@unifi.it (S.T.); giuseppe.gasparro@unifi.it (G.G.); michele.spinicci@unifi.it (M.S.); gianmaria.rossolini@unifi.it (G.M.R.); alessandro.bartoloni@unifi.it (A.B.); lorenzo.zammarchi@unifi.it (L.Z.); 2 Infectious and Tropical Diseases Department, Careggi University Hospital, 50134 Florence, Italy; malcontentic@aou-careggi.toscana.it (C.M.); pozzim@aou-careggi.toscana.it (M.P.); corsip@aou-careggi.toscana.it (P.C.); 3 Clinical Microbiology and Virology Unit, Careggi University Hospital, 50134 Florence, Italy; colaog@aou-careggi.toscana.it

**Keywords:** neglected tropical diseases, HIV, migrants, Europe, Chagas disease, strongyloidiasis, schistosomiasis

## Abstract

Background: Migration phenomena from low- to high-income countries have been constantly increasing in the past years, and migrants living with HIV (MLHIVs) currently represent a non-negligible proportion of the population living with HIV in the European setting. When taken into care, MLHIVs should be screened for tropical diseases capable of asymptomatically persisting for years and leading to severe and/or chronic complications, especially in immunocompromised populations. Methods: We conducted a retrospective study aimed at analyzing the screening ratios and seroprevalences of strongyloidiasis, schistosomiasis, and Chagas disease among MLHIVs who attended a tertiary care hospital outpatient clinic in Florence, Italy. Results: Between 2014 and 2023, 251 MLHIVs were enrolled, of which 85 (33.9%), 137 (54.6%), and 219 (87.3%) were at risk for schistosomiasis, Chagas disease, and strongyloidiasis, respectively. Among individuals at risk for each of these parasitic diseases, 43.4% were screened for strongyloidiasis, 25.9% for schistosomiasis, and 65.0% for Chagas disease, with a seroprevalence of 5.3%, 13.6%, and 0%, respectively. Conclusions: While confirming the relevant burden of neglected parasitic diseases in the MLHIV population, our results suggest the need to improve awareness of these diseases even in the specialist context in order to reduce underdiagnoses and the risk of severe complications, especially in particularly vulnerable groups of the population.

## 1. Introduction

Over the past few decades, migration from low- and middle-income countries (LMICs) to high-income settings has been steadily increasing worldwide. In Europe, 20 million migrants were recorded in 1990, reaching up to 40 million in 2020 [1]. According to Eurostat, more than 5 million people arrived in Europe in 2022, mostly coming from Asia, followed by Africa, Latin America and the Caribbean, North America, and Oceania. Moreover, 1.3 million undocumented migrants were recorded in Europe in 2023. The most popular countries of destination are represented by Germany, Spain, Italy, Czechia, and France [2,3]. Non-EU migrants’ access to care varies among different European countries. While documented migrants are usually entitled to access public health services in the same terms as they are offered to the national population, undocumented migrants do not commonly have access to care, with exceptions made for special conditions (children and pregnancy) and emergencies, although this aspect is very heterogeneous throughout European countries [4,5].

Migrants living with HIV (MLHIVs) represent a relevant population group in Europe, accounting for 48% of new HIV diagnoses in 2022 [6]. In Italy, 31.2% of new HIV diagnoses were made in migrants in the same year [7]. As recommended by national and international guidelines, a broad evaluation of serological status for infectious diseases should be performed in every individual with a new diagnosis of HIV infection. Moreover, a significant proportion of MLHIVs come from areas that are endemic for several parasitic infections. In these cases, tropical diseases capable of persisting for years in an asymptomatic or paucisymptomatic form (e.g., strongyloidiasis, schistosomiasis, and Chagas disease) need to be included among routine initial screenings due to their risk of progression and/or reactivation, possibly leading to severe presentations, especially in the immunosuppressed population [8].

Chagas disease (CD) affects 6–7 million people worldwide. Being endemic in 21 countries of continental Latin America, migration phenomena and the perpetuation of mother-to-child transmission, in the absence of widely implemented screening programs, are also favoring its spread in non-endemic settings [9]. It is estimated that 68.000–123.000 people are currently infected with *Trypanosoma cruzi* in Europe, with only <4% of them being actually diagnosed [10]. In individuals with HIV, especially in cases of severe immunosuppression, *T. cruzi* infection can reactivate, often determining severe central nervous system and heart involvement, with mortality rates as high as 70% [11].

Strongyloidiasis can persist life-long in the host, often being asymptomatic or causing only mild non-specific gastrointestinal, dermatological, or respiratory symptoms in immunocompetent individuals. However, in cases of immunosuppression, such as in the case of HIV/AIDS, the infection can result in life-threatening disseminated disease [12,13,14]. 

Schistosomiasis can cause lifelong infections, during which the adult *Schistosoma* spp. worms release ova that cause chronic inflammation in the genito-urinary system or the gastrointestinal and hepatosplenic system. Chronic inflammation can lead to several structural and functional complications, depending on the most affected structures (i.e., complicated genito-urinary and hepatosplenic schistosomiasis), as well as neoplastic evolution (especially bladder cancer). A correlation between HIV infection and schistosomiasis has been observed. A systematic review conducted by Patel et al. reported a significant association of female genital schistosomiasis in women with HIV, suggesting that this parasitosis could represent a risk factor for acquiring HIV infection [15]. Moreover, schistosomiasis can increase the risk of mother-to-child transmission of HIV, and treatment of schistosomiasis in individuals with HIV showed a favorable impact on decreasing the HIV viral load and slowing down the CD4+ T cells’ decline, both in those receiving and not receiving antiretroviral therapy (ART) [16,17].

Despite several national and international guidelines indicating parasitic disease screenings for migrants coming from endemic areas, data on the prevalence of these neglected tropical diseases (NTDs) in MLHIVs are scant in the European context, and no screening for these infections is widely and systematically implemented [8,18,19,20]. 

We present a 10-year-long observational study conducted on MLHIVs followed in an outpatient clinic (Careggi University Hospital, Florence, Italy). The principal aim of this study was to assess the percentage of serological screening (i.e., screening ratio) for CD, strongyloidiasis, and schistosomiasis in the MLHIV population during routine management in our outpatient clinic, independently of symptoms’ presence. The second aim of this study was to assess schistosomiasis, strongyloidiasis, and CD seroprevalence in the cohort of screened MLHIVs. This study focused on strongyloidiasis, schistosomiasis, and CD due to their relatively high prevalence compared with other NTDs among migrants in the European setting [21,22] and to their specifically relevant impact in the MLHIV population, as mentioned above.

## 2. Materials and Methods

### 2.1. Study Setting, Design, Aims, and Population

Italy is a major European entry point for migrants. In January 2022, over 3.5 million legally residing non-EU citizens were present in this country. Florence surpasses the Italian average, with a non-EU resident percentage of 10.1%, of which 6.8% are from Peru [23].

We conducted an observational, cross-sectional, monocentric study. Migrants with HIV taken into care from January 2014 to December 2023 at the Infectious and Tropical Diseases Unit of Careggi University Hospital, Florence, Italy, were enrolled. Currently, 1800 subjects living with HIV are followed in this unit.

This study involved a historical cohort, of which data have been continually collected and updated since 2014, in order to gather information on different demographic, clinical, and social aspects of MLHIVs that are taken into care in this tertiary healthcare center.

By medical record consultations, we collected information about demographic data, the date of the first infectious disease evaluation, timing, type, and the results of eventually performed serological tests for the three studied NTDs.

The inclusion criteria were as follows:

- Age ≥ 18 years;
- HIV infection confirmed with serological and molecular methods in our laboratory;
- Individuals born in countries outside Italy but living in Italy for at least six months.

The enrolment was not dependent on formal documentation and immigration status.

### 2.2. Migration Policies in Italy

According to the 2023 Italian guidelines for management of migrant health and infectious disease screening at arrival, the path of an extra-European migrant without a visa within the Italian state is divided into a first rescue phase, a first reception phase, and a second reception phase, with national infrastructures present throughout the territory. Some centers, denominated as CAS (extraordinary reception center), are reserved in particular for migrants seeking asylum or considered refugees; other centers, denominated as SAI (integrated reception system), are intended for people with identified vulnerabilities, aiming to start an integration process. From an administrative point of view, a person who enters can request a residence permit or a request for international protection, requests that allow them to have a health card and registration in the national health system. In case of irregular migrants or expired permits, following a declaration of indigence, the person can come into possession of a Foreigner Temporarily Present (STP) code. This code guarantees urgent health services free of charge. It is valid throughout the Italian national territory and is renewable every 6 months [24].

### 2.3. Definitions

Screening ratio: The proportion of performed serological tests over the number of MLHIVs coming from endemic countries was calculated. Reasons for not performing the screening were mainly related to clinical discretion, since there is no standardized screening protocol in our center, and no resource limitation affects the possibility of performing this type of screening. Neglected tropical disease screening to be performed according to nationality was defined based on the World Health Organization (WHO), the Pan American Health Organization (PAHO), the Centers for Disease Control (CDC) guidelines, and the available literature (Supplementary Materials; Table S1) [9,25,26,27,28]. Of note, the indication for strongyloidiasis screening was considered for countries with a seroprevalence of >5% [28]. 

Information regarding the setting (routine clinical practice or during enrolment into clinical studies) in which the screening was required was also collected. In the case of a test performed both for routine clinical practice and in a clinical study setting, we considered only the first case.

Seroprevalence: The seroprevalence of schistosomiasis, strongyloidiasis, and CD within the overall screened population, as well as for each continent of origin, was calculated as the proportion of positive serological results over the overall number of performed tests. In the case of positive serology, clinical, laboratory, treatment, and outcome data for each specific infection were collected.

STP and undocumented migrants: Extra-European migrants who entered Italy illegally or whose entry or residence permit has expired can request an STP code. We defined undocumented migrants as all those MLHIVs in this latter condition as the baseline. 

### 2.4. Serological Screening Assays

*Schistosoma* spp. serology was performed using the commercial kit SCHISTO II Western Blot (WB) IgG^®^ (LDBIO, Lyon, France), according to the manufacturer’s instructions. *Strongyloides* IgG was tested using the enzyme immunoassay *Strongyloides ratti* IgG ELISA^®^, from Bordier (Chatanerie, Switzerland). CD screening was performed with the Chemiluminescent Microparticle Immunoassay ARCHITECT Chagas^®^ (CMIA) from Abbott Laboratories, Wiesbaden, Germany. In the case of a positive serological test, a second test based on crude antigens (Ortho *T. cruzi* enzyme-linked immunosorbent assay (ELISA), from Ortho Clinical Diagnostics, Raritan, Franklin Township, NJ, USA) was automatically performed to confirm the result. In case of discordant results, a third immunoblot test, which used an exoantigen fraction from trypomastigote forms of *T. cruzi* (Chagas Western Blot IgG Assay^®^, LDBio Diagnostics, Lyon, France), was performed.

### 2.5. Statistical Analysis

A descriptive analysis was employed to illustrate the demographic and clinical characteristics of the population. Differences in categorical variables were evaluated with X2/Fisher’s exact test; the Wilcoxon signed-rank test was employed for continuous variables. STATA v18.0 (STATACorp, College Station, TX, USA) was used for statistical analyses. Data were collected using REDCap 8.11.6 (Project REDCap, Nashville, TN, USA). A *p*-value of <0.05 was considered statistically significant.

## 3. Results

**Figure 1:**
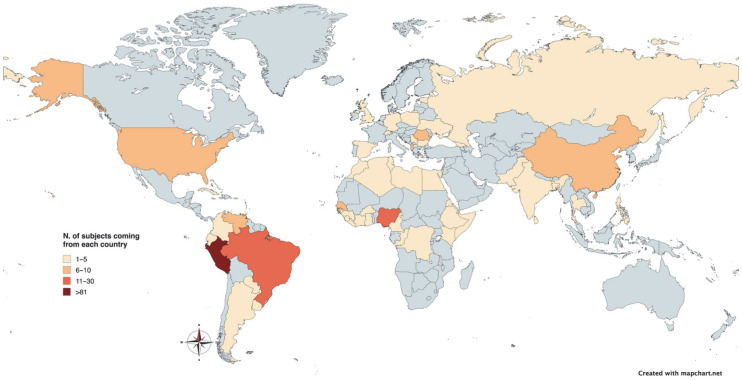
Distribution of enrolled participants by country of origin.

During the study period, 251 MLHIVs were enrolled: 54 (21.5%) were cisgender women, 116 (46.2%) were cisgender men, and 81 (32.3%) were transgender women. The median age was 33.1 years [IQR: 26.7–43.0]. Among them, 139 (55.4%) were born in Latin America, 49 (19.5%) in Africa, 34 (13.6%) in Europe, 21 (8.4%) in Asia, 7 (2.8%) in North America, and 1 (0.4%) in Oceania. The distribution by country of origin is represented in Figure 1.

In total, 105 (41.8%) participants were ART-naïve when taken into care in our center, and 146 (58.2%) were ART-experienced. Diagnosis of HIV infection was made in Italy in 135/251 participants (53.8%).

A history of at least one AIDS-defining event was observed in 43 (17.1%) participants. The median CD4+ lymphocyte count nadir was 448.5 [IQR: 229–641] cell/mm^3^ in MLHIVs with no history of AIDS-defining events and 96.5 [IQR: 12–328] in those with a history of at least one AIDS-defining event. 

The demographic and clinical characteristics of the enrolled population are described in Table 1.

A total of 85 (33.9%) participants were born in countries that are endemic for schistosomiasis, 137 (54.6%) in countries endemic for CD, and 219 (87.3%) came from countries where *Strongyloides stercoralis* seroprevalence is greater than 5%. Twenty-nine (11.6%) were not born in countries epidemiologically considered at risk for strongyloidiasis, schistosomiasis, or CD. 

Schistosomiasis

Among those who were born in schistosomiasis-endemic countries, *Schistosoma* spp. serology was performed in 22/85 (25.9%). One of these 22 (4.5%) serologies was performed in the setting of an epidemiological study, while the remaining were performed as routine screening. Of the 22 tested participants, positive serology was observed in 3/22 (13.6%), coming, respectively, from Brazil, Egypt, and the Ivory Coast. The median latency between first clinical assessment and screening for schistosomiasis was 56.0 days [IQR: 14–254 days]. All positive individuals were asymptomatic and with a normal eosinophil count; none of them had a positive microscopical examination for *Schistosoma* spp. in urine or stool samples. They were all treated with praziquantel.

Chagas disease

Among MLHIVs coming from CD-endemic countries, *Trypanosoma cruzi* serology was performed in 89 (65.0%): 19/89 (21.3%) tests were performed due to the inclusion of the enrolled participants in local epidemiological studies. All tested individuals presented negative results. Among the 89 MLHIVs at risk for CD, 7 cisgender women in their fertile age (15–45 years) were present; 5 (71.4%) of them were screened for CD. The median latency between the first clinical assessment and screening for CD was 77.5 days [IQR: 7–692 days].

Strongyloidiasis

Among MLHIVs born in strongyloidiasis-endemic countries (i.e., with a reported seroprevalence of >5%), *S. stercoralis* serology was performed in 95/219 (43.4%); 2 out of 95 (2.1%) tests were performed in the context of a local epidemiological study. Five out of 95 (5.3%) of the screened participants tested positive, all coming from Peru. The median latency between the first clinical assessment and screening for strongyloidiasis was 54.0 days [IQR: 5–464 days]. All positive individuals were asymptomatic; 3/5 (60.1%) had peripheral eosinophilia. All of them were virologically suppressed and with good immunological status (>500 CD4+ cells/mmc) when diagnosed with strongyloidiasis. Stool culture was positive in one (20.0%); treatment with a single dose of ivermectin was administered to four out of five (80.0%) individuals. One of them was lost at follow-up before ivermectin could be administered. 

The screening ratios and seroprevalences for strongyloidiasis, schistosomiasis, and CD, divided by area of origin, are summarized in Table 2. The screening ratio for strongyloidiasis was significantly higher (70/139, 50.4%) for MLHIVs coming from Latin America compared with other continents of origin (6.3% for Europe, 27.8% for Asia, and 41.3% for Africa). All cases diagnosed with strongyloidiasis upon screening came from Latin America (5/70, 7.1%).

No differences in screening ratios were observed depending on gender (cisgender woman vs. cisgender man vs. transgender woman), country of HIV diagnosis (Italy vs. outside Italy), setting of HIV diagnosis (hospital vs. other settings), or previous AIDS event (Supplementary Materials; Table S2). The screening ratio for strongyloidiasis was significantly higher among MLHIVs with a history of AIDS (61.1%, 22/36) compared with those without a previous AIDS diagnosis (39.0%, 69/177) (*p* = 0.026). The screening ratio for schistosomiasis was significantly higher in those for whom an HIV diagnosis was made in Italy (21/59, 35.6% vs. 1/24, 4.1%); *p* = 0.003). Due to the low number of events, it was not possible to analyze the correlation between seroprevalences and factors such as nadir CD4+ T cell count, previous AIDS-defining events, sex, and immigration status.

**Figure 2:**
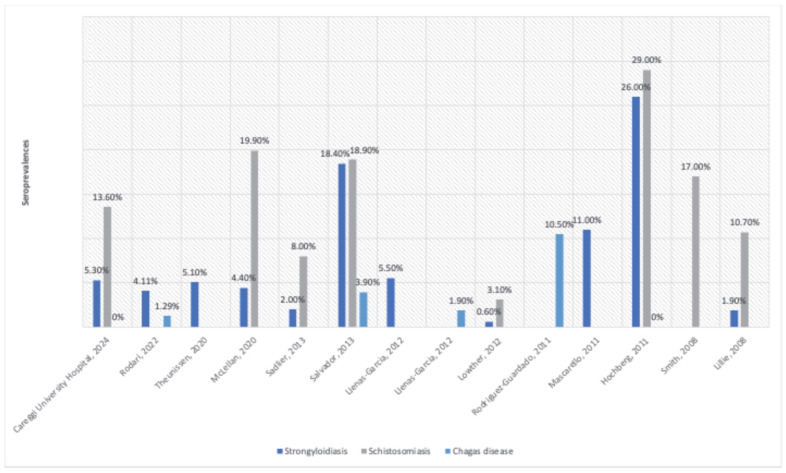
Seroprevalences of strongyloidiasis, schistosomiasis, and Chagas disease in the Careggi University Hospital MLHIV population compared with the available literature data for the MLHIV population [8,19,20,29,30,31,32,33,34,35,36,37,38].

The seroprevalences of strongyloidiasis, schistosomiasis, and CD in the Careggi University Hospital MLHIV population were compared with the available literature data for the MLHIV population [8,19,20,29,30,31,32,33,34,35,36,37,38]. The comparison is presented in Figure 2 and discussed in detail in the Section 4.

## 4. Discussion

This study showed 25.9%, 43.4%, and 60.1% screening ratios and 13.6%, 5.3%, and 0% seroprevalences for schistosomiasis, strongyloidiasis, and CD, respectively, in the study cohort.

The lowest screening ratio (25.9%) but the highest seroprevalence (13.6%) observed for schistosomiasis underline the importance of improving the awareness of schistosomiasis risk in MLHIVs. Serological screening for schistosomiasis should be considered in all migrants from endemic areas, especially in MLHIVs, as genital schistosomiasis can increase HIV sexual transmission by two to three times. Moreover, growing evidence suggests that this infection may raise the risk of mother-to-child HIV transmission [39]. Comparing our results with the available literature, previous studies have reported a schistosomiasis seroprevalence in MLHIVs ranging between 3.1% and 29%, with high variability depending on diagnostic methods and geographic representation in each study population (Figure 2) [8,20,31,32,33,34,35]. While other studies report a higher prevalence among Sub-Saharan African migrants [32,33,34,40], our data showed no significant difference by area of origin, but the low number of cases limits statistical interpretations. Notably, in this study, one of the three individuals with positive *Schistosoma* serology came from Latin America, although screening ratios tended to be lower for Asian and Latin American participants. Despite the highest seroprevalence usually being associated with African origin [31,40], highly endemic hotspots are still present in some Latin American countries, such as Brazil, where our positive case came from. All *Schistosoma* serology-positive individuals were asymptomatic and had a negative microscopical examination for *Schistosoma* spp. in urine or stool samples, supporting the need for screening in all MLHIVs coming from endemic areas, regardless of the presence of suggestive symptoms, which can often be missing in the case of chronic schistosomiasis [32]. Moreover, a lower sensitivity of microscopic methods has been hypothesized for the population with HIV, linked to lower egg excretion in this group [31].

Strongyloidiasis had a slightly higher (43.4%) screening ratio than schistosomiasis, but it is still too low. Although some guidelines recommend strongyloidiasis screening only in case of signs, symptoms, or a laboratory test compatible with active infection [41], this approach would lead to an unacceptably high number of lost diagnoses with a high risk of long-term consequences for asymptomatic, non-diagnosed individuals. Most experts, indeed, support serological screening in asymptomatic individuals according to the epidemiological risk, especially in immunosuppressed populations [42,43,44,45,46]. The observed seroprevalence of 5.3% aligns with the literature data, where seroprevalences range between 0.9% and 26% in MLHIVs [8,19,20,30,31,32,34,35,36,37] (Figure 2). Like those with positive schistosomiasis serology, *S. stercoralis* seropositive participants were also asymptomatic, confirming that nonspecific symptoms such as abdominal pain, diarrhea, or skin manifestations alone are not always associated with strongyloidiasis [32,47,48]. Screening ratios were significantly higher for MLHIVs coming from Africa and Latin America and in those with previous AIDS history, likely reflecting increased clinical concern due to higher risk of reactivation and/or hyperinfection [49]. Coherent with the literature, no significant differences in seroprevalence were found based on area of origin, suggesting that screening attitudes need to be improved regardless of the area of origin, whenever an MLHIV comes from an endemic area [8,40].

Regarding eosinophilia, none of the participants with schistosomiasis had an elevated eosinophil count, whereas 60.1% of those with strongyloidiasis had eosinophilia at the time of diagnosis. While the low number of cases in this study does not allow for analyzing any correlation between eosinophilia and infection risk, and despite some reports demonstrating a significant correlation between strongyloidiasis and eosinophilia [18,32], some studies have previously found that eosinophilia can be less pronounced in immunosuppressed people or may be absent in long-term chronic infections [50]. Although eosinophilia can represent a useful indicator for schistosomiasis or strongyloidiasis, screening should not rely solely on eosinophil levels.

CD screening ratios have reached more acceptable, but still improvable, levels (65.0%). National and international guidelines recommend screening for individuals coming from endemic countries, with a stronger recommendation for the immunosuppressed [51]. In this study, screening ratios were significantly higher for women in their fertile age, a key group to be addressed in order to reduce the risk of mother-to-child transmission, especially in non-endemic countries [52]. Indeed, appropriate treatment before pregnancy significantly reduces the risk of transplacental transmission [53]. No cases of positive *T. cruzi* serology were reported in this study, but this may be related to the predominance of Peruvian participants in our Latin American cohort, a population with generally lower seroprevalence. Studies on the general immigrant population worldwide yielded seroprevalence rates between 1% and 34%, while studies on the MLHIV population reported seroprevalences ranging between 0% and 10.5%, with high variability depending on the country of origin of the tested population (Figure 2) [8,29,32,36,38]. Lower rates in MLHIVs compared with the general migrant population might reflect differences in HIV prevalence in their home country and in risk behaviors adopted in the destination country.

Research programs contributed to the rates of 4.5%, 2.1%, and 21.3% of strongyloidiasis, schistosomiasis, and CD screening, respectively, therefore proving crucial in improving CD screening ratios. It is worth mentioning that one CD case was, indeed, tested for the first time and, consequently, diagnosed with CD in 2021, in the context of an epidemiological study on NTDs in immunosuppressed individuals, despite having previously been in care in our center for 23 years. The subject was diagnosed with HIV before 2014 and, therefore, was not included in the study population. Although screening strategies for the MLHIV population would need a systematic implementation in the European health system, these results highlight the role of scientific projects in raising awareness and toward NTDs screening and detecting cases that routine clinical practice might overlook [37].

Although statistical significance was only observed for strongyloidiasis, screening ratios tended to be higher for the three NTDs in MLHIVs with AIDS history (Supplementary Materials; Table S2); previous studies support that these parasitoses could be more frequent in MLHIVs with uncontrolled HIV, although evidence remains limited [31]. Moreover, advanced HIV disease often overlaps with poor healthcare access, underlining the vulnerability of some MLHIVs and emphasizing the need for more robust screening efforts.

Beyond bringing evidence on the prevalence of schistosomiasis, strongyloidiasis, and CD in MLHIVs, this study also sheds light on current NTDs screening practices in this population. While data on the prevalence of these NTDs in the MLHIV population are scant in Europe, information on screening coverage is even more limited [18,20,30]. To our knowledge, this kind of data has not been reported in Italy before. Considering that high rates of underdiagnosis are estimated in Europe, quantification of missed screening opportunities would help raise awareness about this subject.

The MLHIV population is considered particularly vulnerable, facing both system-associated barriers (e.g., limited access to HIV prevention services, lack of health insurance, lack of social and cultural support, and irregularity on national soil) and personal challenges (e.g., stigma linked to HIV infection, the disease not being entirely understood, language barriers, and family separation) [54,55], which often lead to important delays in HIV diagnosis, poorer clinical outcomes, and high rates of loss to follow-up (up to eight times more with respect to the Italian population) [54]. A recent study conducted in our center reported an 8-year loss to follow-up rate of 8.96 per 100 MLHIVs per year, with higher rates in undocumented migrants [56]. The fragmented status of the reception system probably contributes to this high rate. A significant proportion of participants in our study was composed of sex workers (29.6%) and transgender women (32.3%), who represent a particularly vulnerable population, experiencing even more significant barriers to healthcare access compared with other MLHIVs due to stigma and discrimination [57]. Furthermore, as shown in this study, this population is predominantly young (with a median age of 33.1 years), with a lifelong risk of reactivation or chronic complications caused by schistosomiasis, strongyloidiasis, and CD.

As ART improves life expectancy, and comorbidities emerge, including those associated with immunosuppressive conditions, early diagnosis of NTDs has a pivotal role in improving the health condition of this population group.

Within the limits of this study, the low sample size did not allow us to analyze a statistically significant associations between potential risk factors and higher seroprevalences for the three NTDs. Given the highly variable geographic origin of the included participants, it was not possible to calculate seroprevalences based on country but only based on the continent of origin. These factors certainly give an imprecise picture of the areas with higher seroprevalences for the MLHIV population, as reflected by the high heterogeneity of the results reported in the available literature. In addition, screening was solely based on serology, independently of CD4+ counts; this could have determined an underestimation of the actual prevalence of the three NTDs, especially in cases of low CD4+ T cell counts [31]. In this perspective, future research should evaluate the role, feasibility, and cost-effectiveness of both presumptive NTD treatment and direct parasitological assays as first-line screening approaches in this specific group. Another limitation of this study was the analysis of screening coverage only based on the country of origin, without considering the amount of time spent in the country of origin, nor the migratory route before reaching Italy. Due to the long time period and the retrospective design of this study, data on migratory routes were not homogeneously reported in digital clinical charts; therefore, information on this aspect was neither sufficient nor reliable enough to be considered for the analysis.

Finally, it is important to point out that although schistosomiasis, strongyloidiasis, and CD are among the most common and impactful NTDs in migrant populations, it is equally important to adopt an individualized approach when taking into care MLHIVs, to also identify and appropriately manage other NTDs that may be present, according to more specific epidemiological risk based on past travel, at-risk behaviors, and clinical history.

## 5. Conclusions

Schistosomiasis, strongyloidiasis, and CD, being the three most common NTDs among migrants in Italy, represent a relevant, but often overlooked, health concern in MLHIVs. Implementation of standardized screening protocols in the European context is needed to improve screening coverage and guide health professionals in order to reduce the risk of underdiagnoses and severe complications. Considering the high risk related to disease complications and reactivations, together with the availability of highly effective diagnostic and therapeutic strategies, higher awareness is needed around the importance of screening these NTDs in a particularly vulnerable group of the migrant population.

## Figures and Tables

**Table 1:** Demographic and clinical data of migrants living with HIV taken into care in Careggi University Hospital outpatient clinic from 2014 to 2023, according to epidemiological risk for Chagas disease, schistosomiasis, and strongyloidiasis.

	General Population (*n* = 251)	At Risk for CD (*n* = 137)	At Risk for Sc (*n* = 85)	At Risk for St (*n* = 219)
Cisgender men, n (%)	116 (46.2)	47 (34.3)	43 (50.6)	94 (43.0)
Transgender women, n (%)	81 (32.3)	80 (58.4)	15 (17.7)	81 (37.0)
Median age [IQR]	33.1 [26.7–43.0]	31.9 [26.5–39.3]	35.9 [26.8–45.1]	32.7 [26.1–41.6]
Regular migrants ^1^, n (%)	150 (59.8)	49 (35.8)	66 (77.7)	120 (54.8)
Naïve, n (%)	105 (41.8)	49 (35.8)	36 (42.4)	92 (42.0)
Sex workers, n (%)	68 (29.6)	65 (50.0)	9 (12.5)	68 (34.2)
HIV diagnosed in Italy, n (%)	135 (53.8)	53 (38.7)	59 (69.4)	117 (53.4)
Previous AIDS-defining condition, n (%)	43 (17.1)	19 (13.9)	19 (22.35)	36 (16.4)
Median nadir CD4+ count (cell/mmc) [IQR]	402 [157–595]	402 [193–633]	448.5 [210–619]	410 [169–596]

^1^ Regular in case of fiscal code, and undocumented in case of STP (foreigner temporarily present). CD: Chagas disease; IQR: interquartile range; Sc: schistosomiasis; St: strongyloidiasis.

**Table 2:** Screening ratios and seroprevalences divided by pathogen and area of origin. Bold values indicate statistically significant *p*-values.

	Europe	Asia	Latin America	Africa	Total	*p*-Value
	Screening ratio					
*Strongyloidiasis*	1/16 (6.3)	5/18 (27.8)	70/139 (50.4)	19/46 (41.3)	95/219 (43.4)	**0.003**
*Schistosomiasis*	-	1/8 (12.5)	4/28 (14.3)	17/49 (34.7)	22/85 (25.9)	0.096
*Chagas disease*	-	-	89/137 (65.0)	-	89/137 (65.0)	-
	Seroprevalence					
*Strongyloidiasis*	0/1 (0.0)	0/5 (0.0)	5/70 (7.1)	0/19 (0.0)	5/95 (5.3)	0.597
*Schistosomiasis*	-	0/1 (0.0)	1/4 (25.0)	2/17 (11.8)	3/22 (13.6)	0.724
*Chagas disease*	-	-	0/89 (0.0)	-	0/89 (0.0)	-

## Data Availability

The data are available from the authors upon reasonable request.

## Supplementary Materials

The following supporting information can be downloaded at https://www.mdpi.com/article/10.3390/tropicalmed10100294/s1. Table S1: List of countries considered endemic for each of the three studied NTDs (or with endemicity > 5% for strongyloidiasis), based on World Health Organization (WHO), Pan American Health Organization (PAHO), Centre for Disease Control (CDC) guidelines, and available literature [9,25,26,27,28]. Table S2: Screening ratio for Chagas disease, schistosomiasis, and strongyloidiasis of MLHIVs, divided according to possible factors influencing screening requests.
