# Supplementary material for: Serology-Based Screening and Prevalence of Schistosomiasis, Strongyloidiasis, and Chagas Disease in Migrants Living with HIV: Results from a 10-Year Retrospective Cohort in an Italian Tertiary Healthcare Center
Source: Trop Med Infect Dis. 2025 Oct 16;10(10):294. doi: 10.3390/tropicalmed10100294 (PMC12567673; doi:10.3390/tropicalmed10100294)
Supplement: Supplementary file 1 [file tropicalmed-10-00294-s001.zip › tropicalmed-3811914-supplementary.pdf]

Supplementary material

Table S1. List of countries considered endemic for each of the three studied NTDs (or with endemicity >5% for strongyloidiasis), based on World Health Organization (WHO), Pan American Health Organization (PAHO), Centre for Disease Control (CDC) guidelines, and available literature [9], [25–28].

Chagas disease	Strongyloidiasis	Schistosomiasis
Argentina	Albania	Algeria
Belize	Algeria	Angola
Bolivia	Angola	Argentina
Brazil	Argentina	Benin
Chile	Bangladesh	Botswana
Colombia	Belize	Brazil
Costa Rica	Benin	Burkina Faso
Ecuador	Byelorussia	Burundi
El Salvador	Bolivia	Cambodia
French Guyana	Bosnia	Cameroon
Guatemala	Botswana	Cape Verde
Guyana	Brasile	Chad
Honduras	Bulgaria	China
Mexico	Burkina Faso	Comoros
Nicaragua	Burundi	Congo
Panama	Cambogia	Djibouti
Paraguay	Cameroon	Egypt
Peru	Cape Verde	Eritrea
Suriname	Central Africa Republic	Ethiopia
Uruguay	Chile	Philippines
Venezuela	Chad	Gabon
	China	Gambia
	Colombia	Ghana
	Comoros	Guinea
	Congo	Guinea Bissau
	Costa Rica	Equatorial Guinea
	Croatia	Indonesia
	Cuba	Iran
	Cyprus	Ivory Coast
	Czeck Republic	Kenya
	Dominican Republic	Laos
	Djibouti	Lesotho
	Ecuador	Liberia
	Egypt	Libia
	El Salvador	Madagascar
	Equatorial Guinea	Malawi
	Eritrea	Mali
	Estonia	Mauritania
	Ethiopia	Mauritius
	French Guyana	Morocco
	Gabon	Mozambique
	Gambia	Namibia
	Ghana	Nigeria
	Guadeloupe	Oman
	Guatemala	Puerto Rico
	Guinea	Central Africa Republic
	Guinea Bissau	Rwanda
	Guyana	Sao Tome
	Haiti	Saudi Arabia
	Hungary	Senegal
	India	Seychelles

Indonesia
Iran
Ivory Coast
Jamaica
Japan
Kenya
Kosovo
Laos
Latvia
Lesotho
Liberia
Libia
Lithuania
Macedonia
Madagascar
Malawi
Maldive
Malesia
Mali
Malta
Mauritania
Mauritius
Mexico
Montenegro
Morocco
Mozambique
Myanmar
Namibia
Nepal
Nicaragua
Nigeria
Oman
Pakistan
Panama
Papua New Guinea
Paraguay
Peru
Philippines
Polonia
Puerto Rico
Romania
Russia
Rwanda
Sao Tome
Saudi Arabia
Senegal
Serbia
Seychelles
Sierra Leone
Siria
Slovakia
Slovenia
Somalia
Sri Lanka
South Africa
Sudan
Suriname
Swaziland

Sierra Leone
Siria
Somalia
South Africa
Sudan
Suriname
Swaziland
Tanzania
Togo
Uganda
Venezuela
Yemen
Zambia
Zimbabwe

Thailand
Tanzania
Togo
Turkey
Uganda
Ukraine
Uruguay
Venezuela
Vietnam
Yemen
Zambia
Zimbabwe

Table S2. Screening ratio for Chagas disease, schistosomiasis, and strongyloidiasis of MLHIVs, divided according to possible factors influencing screening request.

	**MLHIV at risk for CD (n=137), subjects screened**		**MLHIV at risk for Sc (n=85), subjects screened**		**MLHIV at risk for St (n=219), subjects screened**	
	n, (%)[1]	*p*-value	n, (%)[1]	*p*-value	n, (%)[1]	*p*-value
Cisgender men	31 (66.0)	0.938	12 (28.0)	0.467	39 (41.5)	0.086
Cisgender women	6 (60.0)		8 (30.0)		14 (31.8)	
Transgender women	52 (65.0)		2(13.3)		42 (51.9)	
HIV diagnosed in Italy	36 (67.9)	0.765	21 (35.6)	**0.003**	47 (40.1)	0.172
HIV diagnosed abroad	53 (65.4)		1 (4.17)		48 (49.5)	
HIV diagnosed in hospital	15 (65.2)	0.991	9 (36.0)	0.251	25 (49.0)	0.364
HIV diagnosed in other settings	69 (65.9)		13 (23.6)		66 (41.8)	
Past AIDS diagnosis	14 (73.7)	0.507	6 (31.6)	0.569	22 (61.1)	**0.026**
AIDS never diagnosed	71 (62.8)		15 (23.4)		69 (39.0)	
Regular[2]	29 (59.2)	0.290	18 (27.3)	0.585	45 (37.5)	0.053
Undocumented[2]	60 (68.2)		4 (21.1)		50 (50.5)	
ART-Naïve	33 (67.4)	0.663	10 (27.8)	0.732	36 (39.1)	0.280
ART-Experienced	56 (63.6)		12 (24.5)		59 (46.5)	

[1] The percentage is calculated on the total of the single subgroup (e.g. in "Cisgender men, cisgender women and transgender women" the first, second and third percentages are calculated on the total number of cisgender men, cisgender women, and transgender women included in the corresponding at-risk population);
[2] Regular in case of fiscal code, undocumented in case of STP (foreigner temporarily present).
ART: antiretroviral therapy; CD: Chagas disease; MLHIV: migrants living with HIV; Sc: schistosomiasis; St: strongyloidiasis.
Values in bold are statistically significant
